# Non-metric traits of the cranium: are they related one with each other? A novel approach to skeletal variants

**DOI:** 10.1007/s00414-026-03783-4

**Published:** 2026-03-30

**Authors:** Andrea Palamenghi, Sofia Alemanno, Antonio José Aragon Molina, Giulia Caccia, Michaela Cellina, Salvatore Cappabianca, Alfonso Reginelli, Ferdinando Caranci, Ruggero Donida Labati, Fabio Scotti, Vincenzo Piuri, Debora Mazzarelli, Annalisa Cappella, Carlo Pietro Campobasso, Cristina Cattaneo, Danilo De Angelis, Daniele Maria Gibelli

**Affiliations:** 1 Laboratorio di Antropologia e Odontologia Forense (LABANOF), Sezione di Medicina Legale, Dipartimento di Scienze Biomediche per la Salute, Università degli Studi di Milano, Milan, Italy; 2 Dipartimento di Scienze Biomediche per la Salute, Università degli Studi di Milano, Milan, Italy; 3 Department of Experimental Medicine, University of Campania “Luigi Vanvitelli”, Naples, Italy; 4 Department of Computer Science, Università degli Studi di Milano, Milan, Italy; 5 Unit of Radiology, Fatebenefratelli Hospital, ASST Fatebenefratelli Sacco, Milan, Italy; 6 Unit of Radiology, “Luigi Vanvitelli” University Hospital, Naples, Italy; 7 Laboratory of Applied Human Morphology, IRCCS Policlinico San Donato, Milan, Italy

**Keywords:** Non-metric traits, anatomical variants, cranium, biological anthropology, forensic anthropology, CT-scan, personal identification

## Abstract

Non-metric traits are anatomical variants of the human skeleton that are commonly used as morphological descriptors and have shown potential for personal identification. Although their frequencies are reported for different contemporary and archaeological populations, very little is known about their origins and even less is reported about their reciprocal correlations.

This study aims at assessing the possible correlation among 21 different non-metric traits of the cranium (12 pair and symmetric, 9 unpaired and median) to verify if they are correlated one with each other. The variants were observed on 733 CT-scan collected from the radiological databases of two Italian hospitals. A pairwise correlation analysis among features was performed using Pearson’s correlation index (|r|).

Results show that only four significant positive correlations were found, namely between number of septa and scallops in the frontal sinuses, between the pneumatization of pterygoid processes and greater wings bilaterally, and between the pneumatization of greater wings and clinoid processes on the left side with a |r| between 0.333 and 0.650. The single negative correlation was between metopism and number of scallops in the frontal sinuses with a |r| of -0.408 (*p* < 0.05).

This study shows that most of anatomical variants are largely independent one from the others: this novel information represents a starting point for an in-depth study on the origin of non-metric traits and confirms their potential for personal identification.

## Introduction

In biological anthropology, non-metric traits represent skeletal variants, qualitatively assessable and lacking a Mendelian transmission [1]; for this reason, some authors refer to them as “discrete”, “discontinuous or quasi-continuous” or “epigenetic”, although their origin is far from being clarified, especially for what concerns the respective contribution of genes and environment. For more than two centuries these variants have been considered mere skeletal anomalies and most of them are commonly used as general descriptors in the analysis of skeletal remains. In bioarchaeology, the presence of the same non-metric traits in different skeletons is considered a hint of their common familiar groups, although their heritability is highly debated [2]. In the last years non-metric traits are gaining a novel importance especially for the chance of classifying human remains for identification purposes.

In forensic anthropology, non-metric traits may be used as individualizing features in personal identification of human remains, which represents the goal of a death investigation, with crucial legal and ethical implications [3, 4]. Personal identification is a comparative scientific procedure between biological details obtained from the remains, i.e., postmortem (PM) data, and information related to missing persons (antemortem, AM) with a similar biological profile (sex, population affinity, age-at-death and stature) to the unidentified remains [5]. While conventional primary identifiers (i.e., DNA, fingerprints and odontological features) are the most reliable to provide identification [6, 7], in some instances they may be inconsistent or unavailable. Such limitations have been documented across a wide range of contexts, including routine forensic casework, humanitarian investigations, and mass disaster scenarios [8–10]. In these situations, direct biological relatives necessary for DNA comparison be unreachable, unknown or may even refuse to cooperate, as well as access to fingerprints and antemortem dental records may be limited, entirely absent or impossible to obtain. These constraints can significantly hinder the identification process based on primary identifiers and highlight the need for complementary approaches. Indeed, secondary identifiers are increasingly gaining attention as valid markers [11], especially for what concerns skeletal features visible and easily detectable from medical imaging techniques such as conventional X-rays analysis and CT-scan [12–15]. The range of possible identifying features is very large, including morphology of skeletal and dental anatomical details [16–22], and even skin descriptors such as nevi and scars [23–27].

In the last decade, several authors have explored the potential of non-metric traits for personal identification [28–34]. In addition to the assessment of their presence, location and morphology to evaluate possible correspondences between antemortem and postmortem records, these variants allow the anthropologist to apply a statistical framework based on known frequencies within groups. Watamaniuk and Rogers [35]recorded frequencies of morphological traits on thoracic vertebrae from X-rays and suggested their use to calculate the strength of matches and the expected number of people showing a specific pattern of traits in the Identification Universe (i.e., the group of individuals that should represent the unidentified remains, such as a missing person list). Frequencies of multiple traits can be multiplied together via the product rule (compound frequencies) and Likelihood Ratios (LR) can be produced as well to strengthen the observation based on morphological elements and support the identification within a statistical framework, similarly to DNA results [36–38]. Palamenghi et al. applied this approach to different population groups [39, 40]. The results showed strong LR and very low probabilities to find two people sharing the same set of cranial traits, demonstrating the potential of creating a unique pattern or code based on presence or absence of specific non-metric traits, univocally referring to a single individual [40].

However, this approach, although it seems promising, requires the independence of non-metric traits one from each other: in fact, possible correlation, if significant and with a high correlation index, may lead to a repetition of frequencies and the compound frequency [35]. Therefore, the independence of traits should be carefully assessed and proven before using the statistical tools offered by the frequencies of these features.

The present study specifically aims at consolidating the statistical framework for personal identification by systematically investigating reciprocal correlations among cranial non-metric traits in two samples from two Italian cities, assessed through CT-scan examinations. Verifying the assumption of independence is a fundamental step to support the robust application of compound frequencies, while also providing additional insights into the biological origin of these variants.

## Materials and methods

In total, 733 CT-scans were acquired from two Italian hospitals in the cities of Milan (200 CT-scans, 109 from male and 91 from female patients) and Naples (533 CT-scan, 305 from male and 228 from female patients), respectively. The research was conducted in accordance with the Declaration of Helsinki and the study was approved by the local ethical committees of the two hospitals (7331/2019 for Milan, 0001708/I for Naples).

The acquisition protocol included the following settings: 120 kV, 320 mAs, 40 × 0.6 mm collimation, 1-second tube rotation, 1 mm reconstruction thickness, with H21s (smooth) filters for soft tissue and H60 (sharp) filters for bone structures. The scans were primarily performed to investigate suspected cranial fractures in cases of trauma, sinusitis, or neurological conditions. Patients affected by traumatic lesions, congenital or acquired cranial deformities, or diseases affecting the cranium were excluded from the analysis. Overall, the sample included 414 males (mean age: 53.5 ± 19.3 years) and 319 females (mean age: 54.2 ± 19 years).

Each CT-scan was loaded individually on the open-source DICOM visualizer software ITK-SNAP [41] to evaluate the anatomical variants in sagittal, coronal and transverse planes. A set of 21 cranial non-metric traits (12 pair and symmetric, 9 unpaired and median) was assessed and scored as described in Table 1. Eventually, since 12 traits were bilateral, 33 variants were concurrently assessed for each CT-scan by two observers.

**Table 1:** Description and scoring of the cranial non-metric traits included in the study

Trait	Description	Pair and symmetric	Unpaired and median	Scoring
Metopism	Persistent suture along the midline of the frontal bone		X	Present/absent
Number of septa in the frontal sinuses	Number of bony laminae dividing each sinus in pouches		X	Number of septa
Number of scallops in the frontal sinuses	Number of pouches in the superior portion of each sinus		X	Number of scallops
Foramen of Vesalius	Additional foramen anterior to the oval foramen in the medial portion of the greater sphenoid wing	X		Present/absent
Pharyngeal tubercle	Tubercle on the inferior aspect of the pars basilaris of the occipital bone		X	Present/absent
Pharyngeal canal	Complete or incomplete canal on the pars basilaris of the occipital bone		X	Present complete/present incomplete/absent
Supraorbital foramen	Foramen on the superior margin of the orbit instead of the supraorbital notch	X		Present/absent
Number of accessory supraorbital foramina	Accessory foramen close to the supraorbital notch	X		Present/absent
Number of accessory zygomatic foramen	Accessory foramina on the zygomatic bone	X		Present/absent
Nasal septal spur	Bony spur in the nasal septum		X	Present/absent
Palatine torus	Bony protuberance along the median part of the hard palate		X	Present/absent
Maxillary torus	Bony protuberance on the palatal surface of the molar alveoli	X		Present/absent
Pneumatization of crista galli	Pneumatized crista galli of the ethmoid bone		X	Present/absent
Pneumatization of middle turbinate	Pneumatized middle turbinate	X		Present/absent
Paradoxical middle turbinate	Convexity of the middle turbinate laterally oriented	X		Present/absent
Pneumatization of dorsum sellae	Pneumatized dorsum sellae of the sphenoid bone		X	Present/absent
Pneumatization of clinoid processes	Pneumatized clinoid processes of the sphenoid bone	X		Present/absent
Pneumatization of greater wings	Pneumatized greater wings of the sphenoid bone	X		Present/absent
Pneumatization of pterygoid processes	Pneumatized pterygoid processes of the sphenoid bone	X		Present/absent
Lesser palatine foramina	Accessory foramina close to the greater palatine foramina	X		Present/absent
Palatine bridge	Bony spicules partly or completely connecting the surface of palatine bones	X		Present complete/present incomplete/absent

### Statistical analyses

A pairwise correlation analysis between features was performed using Pearson’s correlation coefficient (|r|). Pearson’s correlation coefficient was selected because the dataset includes both binary traits and ordinal/count variables (e.g., number of septa and scallops in frontal sinuses), and a unified correlation framework was preferred for comparability across all traits. The primary objective of this study was to test pairwise independence, which is the critical assumption underlying the product rule and compound frequency calculations used in forensic identification frameworks. Positive correlations imply that both features tend to appear together, while negative correlations indicate that the presence of one feature is associated with the absence of the other. To ensure relevance, the study focused on correlations with an absolute value greater than 0.3, as this threshold helped distinguish meaningful associations from background noise, based on comparisons with randomized data. From a methodological perspective, it is acknowledged that the absence of linear correlation (Pearson’s coefficient) does not strictly imply stochastic independence, unless the variables follow a joint Gaussian distribution. However, given the discrete and predominantly binary nature of non-metric traits, the pairwise correlation analysis represents the most robust proxy to detect dependencies. Consequently, traits showing negligible correlation were considered unlikely to introduce substantial redundancy in compound frequency calculations.

## Results

**Fig. 1:**
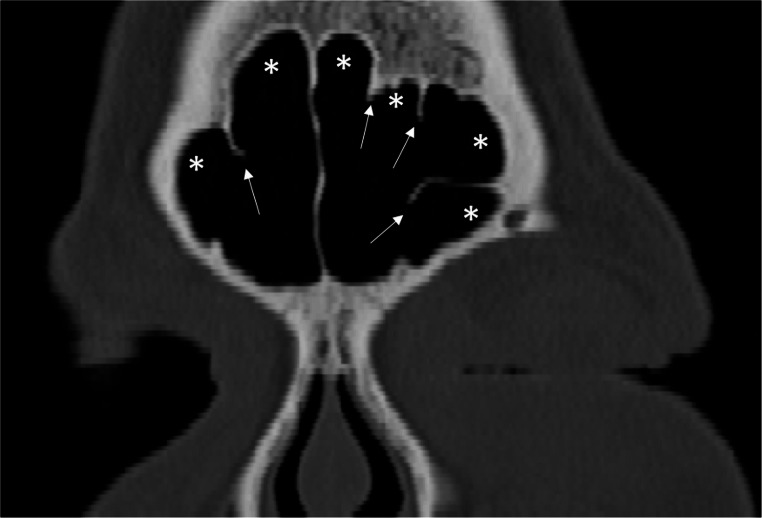
Septa (white arrows) and scallops (white asterisks) of the frontal sinuses

**Fig. 2:**
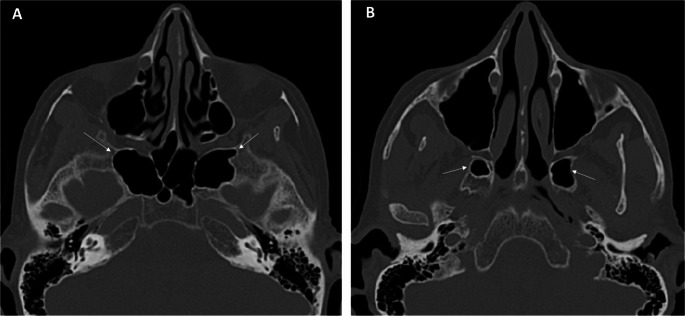
**A** Bilateral pneumatization of the greater wings of the sphenoid bone (white arrows); **B** Bilateral pneumatization of the pterygoid processes of the sphenoid bone (white arrows)

**Fig. 3:**
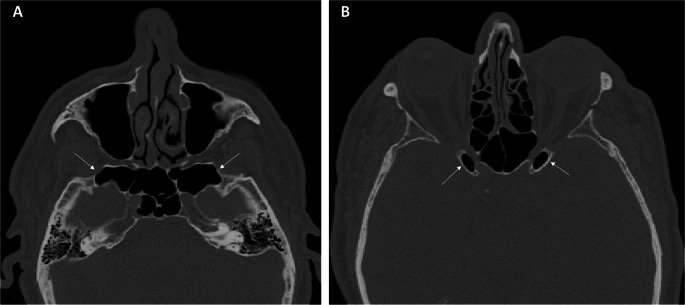
**A** Bilateral pneumatization of the greater wings of the sphenoid bone (white arrows); **B** Bilateral pneumatization of the clinoid processes of the sphenoid bone (white arrows)

**Fig. 4:**
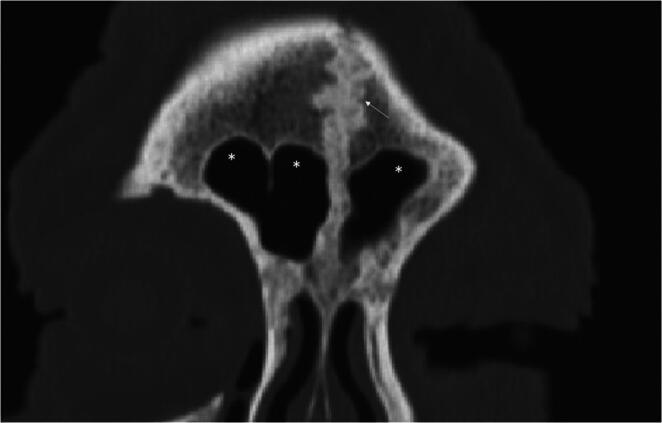
Persistent metopic suture (white arrow) and scallops of the frontal sinuses (white asterisks)

The Milan and Naples samples were grouped together to analyze the significant positive and negative correlations among the variants in the entire dataset. Among all the possible correlations according to 12 pair and symmetric and 9 unpaired and median non-metric traits, only five were found significant (*p* < 0.05); four out of five presented positive correlations, namely between the number of septa and scallops in the frontal sinuses, between the pneumatization of pterygoid processes and greater wings bilaterally, and between the pneumatization of greater wings and clinoid processes on the left side (Figs. 1, 2 and 3) with a |r| between 0.333 and 0.650. Only the correlation between metopism and number of scallops in the frontal sinuses was negative, with a |r| of -0.408 (Table 2; Fig. 4).

**Table 2:** Significant correlations between features in the entire dataset (Table 3)

Trait 1	Trait 2	Correlation
Number of septa in the frontal sinuses	Number of scallops in the frontal sinuses	0.650
Pneumatization of pterygoid processes (left)	Pneumatization of greater wings (left)	0.377
Pneumatization of pterygoid processes (right)	Pneumatization of greater wings (right)	0.355
Pneumatization of greater wings (left)	Pneumatization of clinoid processes (left)	0.333
Metopism	Number of scallops in the frontal sinuses	-0.408

The correlations are confirmed when data are analyzed separately according to sex, except for the correlation between pterygoid process and the greater wings on the right side which was observed in males, but not in females. Moreover, a novel correlation was found between the pneumatization of the greater wings and the clinoid processes, but only in males. For positive correlations, |r| ranged between 0.311 and 0.659, whereas for the unique negative correlation (between metopism and scallops of the frontal sinuses) it was -0.438 in males, -0.370 in females (Table 3)

**Table 3:** Significant correlations between features in males and females. NS: not significant correlation

Trait 1	Trait 2	Correlation |*r*|	
		Males	Females
Number of septa in the frontal sinuses	Number of scallops in the frontal sinuses	0.659	0.630
Pneumatization of pterygoid processes (left)	Pneumatization of greater wings (left)	0.429	0.311
Pneumatization of pterygoid processes (right)	Pneumatization of greater wings (right)	0.418	NS
Pneumatization of greater wings (right)	Pneumatization of clinoid processes (left)	0.330	NS
Pneumatization of greater wings (left)	Pneumatization of clinoid processes (left)	0.325	0.349
Metopism	Number of scallops in the frontal sinuses	-0.438	-0.370

The descriptive statistics of each trait considered in the study are reported in Table 4

**Table 4:** Descriptive statistics of the non-metric traits. Number of individuals (N) and related frequencies of each trait

		Males Naples		Males Milan		Females Naples		Females Milan	
Trait	Feature	N	Frequency	N	Frequency	N	Frequency	N	Frequency
Metopism	0	288	0.94	107	0.98	217	0.95	91	1.00
	1	18	0.06	2	0.02	11	0.05	0	0.00
Number of septa in the frontal sinuses	0	74	0.24	13	0.12	60	0.26	11	0.12
	1	65	0.21	22	0.20	48	0.21	32	0.35
	2	52	0.17	34	0.31	61	0.27	24	0.26
	3	55	0.18	31	0.28	33	0.14	18	0.20
	4	35	0.11	5	0.05	14	0.06	3	0.03
	5	16	0.05	2	0.02	7	0.03	1	0.01
	6	6	0.02	1	0.01	3	0.01	1	0.01
	7	3	0.01	0	0.00	0	0.00	0	0.00
	8	0	0.00	1	0.01	2	0.01	1	0.01
Number of scallops in the frontal sinuses	0	17	0.06	2	0.02	8	0.04	1	0.01
	1	7	0.02	2	0.02	7	0.03	0	0.00
	2	31	0.10	1	0.01	28	0.12	1	0.01
	3	44	0.14	4	0.04	36	0.16	9	0.10
	4	44	0.14	14	0.13	55	0.24	16	0.18
	5	57	0.19	23	0.21	47	0.21	18	0.20
	6	41	0.13	20	0.18	25	0.11	17	0.19
	7	32	0.10	15	0.14	15	0.07	14	0.15
	8	25	0.08	15	0.14	5	0.02	8	0.09
	9	6	0.02	4	0.04	0	0.00	3	0.03
	10	1	0.00	6	0.06	1	0.00	3	0.03
	11	1	0.00	2	0.02	1	0.00	1	0.01
	12	0	0.00	1	0.01	0	0.00	0	0.00
Foramen of Vesalius (right)	0	68	0.22	31	0.28	52	0.23	40	0.44
	1	238	0.78	78	0.72	176	0.77	51	0.56
Foramen of Vesalius (left)	0	59	0.19	40	0.37	54	0.24	48	0.53
	1	247	0.81	69	0.63	174	0.76	43	0.47
Pharyngeal tubercle	0	19	0.06	4	0.04	29	0.13	5	0.05
	1	287	0.94	105	0.96	199	0.87	86	0.95
Pharyngeal canal	0 (absent)	257	0.84	83	0.76	201	0.88	71	0.78
	1 (incomplete)	44	0.14	23	0.21	25	0.11	20	0.22
	2 (complete)	5	0.02	3	0.03	2	0.01	0	0.00
Pneumatization of dorsum sellae	0	169	0.55	104	0.95	115	0.50	88	0.97
	1	137	0.45	5	0.05	113	0.50	3	0.03
Supraorbital foramen (right)	0	207	0.68	96	0.88	147	0.64	77	0.85
	1	99	0.32	13	0.12	81	0.36	14	0.15
Supraorbital foramen (left)	0	209	0.68	94	0.86	127	0.56	74	0.81
	1	97	0.32	15	0.14	101	0.44	17	0.19
Number of accessory supraorbital foramina (right)	0	203	0.66	66	0.61	163	0.71	64	0.70
	1	103	0.34	43	0.39	65	0.29	27	0.30
Number of accessory supraorbital foramina (left)	0	205	0.67	62	0.57	153	0.67	54	0.59
	1	101	0.33	47	0.43	75	0.33	37	0.41
Number of accessory zygomatic foramen (right)	0	107	0.35	8	0.07	103	0.45	7	0.08
	1	199	0.65	101	0.93	125	0.55	84	0.92
Number of accessory zygomatic foramen (left)	0	102	0.33	5	0.05	92	0.40	5	0.05
	1	204	0.67	104	0.95	136	0.60	86	0.95
Nasal septal spur	0	179	0.58	69	0.63	130	0.57	54	0.59
	1	127	0.42	40	0.37	98	0.43	37	0.41
Pneumatization of crista galli	0	289	0.94	101	0.93	217	0.95	85	0.93
	1	17	0.06	8	0.07	11	0.05	6	0.07
Pneumatization of middle turbinate (right)	0	269	0.88	80	0.73	177	0.78	69	0.76
	1	37	0.12	29	0.27	51	0.22	22	0.24
Pneumatization of middle turbinate (left)	0	254	0.83	73	0.67	183	0.80	60	0.66
	1	52	0.17	36	0.33	45	0.20	31	0.34
Paradoxical middle turbinate (right)	0	252	0.82	82	0.75	195	0.86	69	0.76
	1	54	0.18	27	0.25	33	0.14	22	0.24
Paradoxical middle turbinate (left)	0	242	0.79	82	0.75	200	0.88	70	0.77
	1	64	0.21	27	0.25	28	0.12	21	0.23
Pneumatization of pterygoid processes (right)	0	258	0.84	88	0.81	186	0.82	71	0.78
	1	48	0.16	21	0.19	42	0.18	20	0.22
Pneumatization of pterygoid processes (left)	0	246	0.80	83	0.76	186	0.82	72	0.79
	1	60	0.20	26	0.24	42	0.18	19	0.21
Pneumatization of greater wings (right)	0	270	0.88	81	0.74	191	0.84	73	0.80
	1	36	0.12	28	0.26	37	0.16	18	0.20
Pneumatization of greater wings (left)	0	258	0.84	83	0.76	186	0.82	72	0.79
	1	48	0.16	26	0.24	42	0.18	19	0.21
Pneumatization of clinoid processes (right)	0	275	0.90	91	0.83	205	0.90	73	0.80
	1	31	0.10	18	0.17	23	0.10	18	0.20
Pneumatization of clinoid processes (left)	0	271	0.89	90	0.83	207	0.91	78	0.86
	1	35	0.11	19	0.17	21	0.09	13	0.14
Lesser palatine foramina (right)	0	23	0.08	5	0.05	13	0.06	9	0.10
	1	283	0.92	104	0.95	215	0.94	82	0.90
Lesser palatine foramina (left)	0	23	0.08	4	0.04	12	0.05	9	0.10
	1	283	0.92	105	0.96	216	0.95	82	0.90
Palatine bridge	0 (absent)	63	0.21	6	0.06	54	0.24	2	0.02
	1 (incomplete)	224	0.73	89	0.82	167	0.73	83	0.91
	2 (complete)	19	0.06	14	0.13	7	0.03	6	0.07
Palatine bridge	0 (absent)	59	0.19	5	0.05	56	0.25	4	0.04
	1 (incomplete)	229	0.75	94	0.86	157	0.69	83	0.91
	2 (complete)	18	0.06	10	0.09	15	0.07	4	0.04
Palatine torus	0	232	0.76	87	0.80	159	0.70	75	0.82
	1	74	0.24	22	0.20	69	0.30	16	0.18
Maxillary torus (right)	0	289	0.94	99	0.91	214	0.94	85	0.93
	1	17	0.06	10	0.09	14	0.06	6	0.07
Maxillary torus (left)	0	291	0.95	98	0.90	217	0.95	86	0.95
	1	15	0.05	11	0.10	11	0.05	5	0.05

## Discussion

Anatomical variants represent the modifications of the body anatomy due to genetic and environmental causes. With time, the concept of anatomical variant has changed, passing from a mere “joke of nature” (*lusus naturae*) [1] to important factors possibly influencing different disciplines, from clinics and surgery to forensic anthropology, although its precise definition is still elusive.

In the field of personal identification of human remains, skeletal and dental morphological features are gaining a growing importance thanks to their high individualizing power [11, 28, 42]. Among these, non-metric traits are included and are worth being explored in depth for their individualizing potential. The value of non-metric traits in the process of personal identification has been extensively demonstrated in a comparative qualitative process [28–33]. In addition, novel statistical approaches can be built upon frequencies of variants providing a code which can support the preliminary search of possible suspects of identity, it can be used in the comparison between antemortem and postmortem data, and it quantifies the individuality of a specific mix of variants [40, 43]. However, this statistical approach requires reciprocal independence of non-metric traits as compound frequency needs to be calculated starting from independent traits [36–38]. Despite this crucial point, the independence of cranial non-metric traits remains a highly debated topic in anthropology Indeed, over the years, several studies have investigated this issue, but the findings have often been inconsistent or inconclusive [1].

The present article aimed at verifying the reciprocal correlation among 21 cranial non-metric traits (12 pair and symmetric and 9 unpaired and median, for a total of 33 features) throughout CT-scan assessment. Results showed that, among the possible 1024 correlations which could be found among all the 33 features, only five showed a significant correlation: this information highlights that non-metric traits seem to be largely independent one from each other. As non-metric traits have combined genetic and environmental causes, one may conclude that these vary according to a single variant, although their precise origin is still unknown. The significant correlations observed are generally weak, as their absolute correlation coefficients remain below 0.7 for positive associations and above − 0.7 for negative ones. This indicates that the relationships between traits are far from deterministic, meaning that the presence or absence of one variant cannot reliably predict the presence or absence of another correlated trait. Such evidence may therefore support the usefulness of compound probability approaches in personal identification, since largely independent traits can increase discriminatory power when considered jointly. Nevertheless, although the correlations appear modest, it is important to stress that pairwise independence constitutes a necessary but not sufficient condition for full joint independence among multiple traits. In other words, even weak dependencies between individual trait pairs may accumulate when several variants are analyzed together, potentially introducing bias into compound frequency estimates. This issue should be carefully evaluated in forensic applications, where accurate probability assessments are essential for reliable identification.

As for the few significant correlations, an explanation can be proposed. For instance, the number of septa clearly influences the number of scallops in the frontal sinuses: an increase in septa naturally results in a greater subdivision of the pneumatized space, thereby producing a higher number of scallops [44]. The correlations between different variants of pneumatization of the sphenoid bone (pterygoid processes and greater wings, greater wings and clinoid processes) can be justified by the same biological pneumatizing processes and the anatomical proximity of its air-filled structures within the same bone. In fact, the tendency of pneumatized areas to expand into adjacent regions may result in developmental overlap, producing the observed associations among these variants [45, 46].

Correlation between persistence of metopic suture and development of frontal sinuses is a highly debated topic in literature: some authors found that metopism is weakly related to the agenesis of frontal sinuses [47], whereas other articles did not find any relationship between the persistence of metopic suture and the size of pneumatized frontal areas [48, 49]. The present article found that the frontal sinuses show less subdivisions in scallops in case of persistent metopic suture, which may be indirectly linked to a small size. However, as metrical measurements were not taken into consideration, no conclusions can be drawn about possible correlations between metopism and size of frontal sinuses.

Finally, the analysis of correlations among non-metric traits produced the same results when males and females were considered separately: this result possibly suggests that the chosen non-metric traits do not show significant differences according to sex, once again stressing their importance for a possible identification procedure.

This article is nevertheless affected by some limitations, such as the origin of CT-scans from two different hospitals: this choice was taken to provide a large number of individuals for statistical analyses. Although the two groups belong to the same country, possible local geographical variations cannot be excluded. The next studies will focus on increasing the sample size of both populations to verify possible geographic differences.

## Conclusions

In the last decades non-metric traits have been recognized as important factors possibly influencing clinics, surgery and pathology. Although their origin remains largely unclear, they may have a potential for personal identification in forensic anthropology, especially throughout frequential approaches aiming at calculating rareness of specific mix of variants and helping comparisons among different codes. However, this approach requires the independence of each variant from the other one. This study showed that the cranial skeletal variants considered are substantially unrelated one with each other, and the few correlations have a weak correlation coefficient. In conclusion, the present study contributes with valuable and original data to the understanding of cranial non-metric traits. In particular, the lack of significant patterns of correlation provides important hints about the development of these variants and strengthens, upon further investigations, their possible application to personal identification.

## Data Availability

The data presented in this study are available from the corresponding author upon reasonable request.

## References

[1] Hauser G, De Stefano F (1989) Epigenetic variants of the human skull. Schweizerbart, Stuttgart

[2] Sjøvold T (1984) A Report on the Heritability of Some Cranial Measurements and Non-Metric Traits. Multivariate Statistical Methods in Physical Anthropology. Springer Netherlands, Dordrecht, pp 223–246

[3] Pilli E, Palamenghi A, Cattaneo C (2025) Forensic skeletal and molecular anthropology face to face: Combining expertise for identification of human remains. Ann N Y Acad Sci. 10.1111/nyas.15398 40637644 10.1111/nyas.15398 PMC12412729

[4] Cattaneo C, De Angelis D, Mazzarelli D, Porta D, Poppa P, Caccia G, D’Amico ME, Siccardi C, Previderè C, Bertoglio B, Tidball-Binz M, Ubelaker D, Piscitelli V, Riccio S (2023) The rights of migrants to the identification of their dead: an attempt at an identification strategy from Italy. Int J Legal Med 137:145–156. 10.1007/s00414-022-02778-1 35277774 10.1007/s00414-022-02778-1 PMC9816290

[5] Ubelaker DH, Shamlou A, Kunkle A (2019) Contributions of forensic anthropology to positive scientific identification: a critical Review. Forensic Sci Res 4:45–50. 10.1080/20961790.2018.1523704 30915416 10.1080/20961790.2018.1523704 PMC6427489

[6] INTERPOL, DISASTER VICTIM IDENTIFICATION GUIDE INTERPOL DVI GUIDE REVIEW SCHEDULE (2023). New INTERPOL DVI Guide. https://www.interpol.int/content/download/589/file/DVI_DVI%20Guide%202023.pdf (last access: November the 18th 2025)

[7] Sweet D (2010) INTERPOL DVI best-practice standards–An overview. Forensic Sci Int 201:18–21. 10.1016/j.forsciint.2010.02.031 20303223 10.1016/j.forsciint.2010.02.031

[8] Mazzarelli D, Milotta L, Franceschetti L, Maggioni L, Merelli VG, Poppa P, Porta D, De Angelis D, Cattaneo C (2021) Twenty-five years of unidentified bodies: an account from Milano, Italy. Int J Legal Med 135:1983–1991. 10.1007/s00414-021-02560-9 33748873 10.1007/s00414-021-02560-9

[9] Gunawardena SA, Samaranayake R, Dias V, Pranavan S, Mendis A, Perera J (2019) Challenges in implementing best practice DVI guidelines in low resource settings: lessons learnt from the Meethotamulla garbage dump mass disaster. Forensic Sci Med Pathol 15:125–130. 10.1007/s12024-018-0033-4 30306346 10.1007/s12024-018-0033-4

[10] Palamenghi A, Cattaneo C (2024) The response of the forensic anthropology scientific community to migrant deaths: Where are we at and where do we stand? Forensic Sci Int 364:112235. 10.1016/j.forsciint.2024.112235 39332311 10.1016/j.forsciint.2024.112235

[11] Blau S, Roberts J, Cunha E, Delabarde T, Mundorff AZ, de Boer HH (2023) Re-examining so-called ‘secondary identifiers’ in Disaster Victim Identification (DVI): Why and how are they used? Forensic Sci Int 345:111615. 10.1016/j.forsciint.2023.111615 36907108 10.1016/j.forsciint.2023.111615

[12] Quatrehomme G, Biglia E, Padovani B, du Jardin P, Alunni V (2014) Positive identification by X-rays bone trabeculae comparison. Forensic Sci Int 245:e11–e14. 10.1016/j.forsciint.2014.09.019 25450510 10.1016/j.forsciint.2014.09.019

[13] Quatrehomme G, Fronty P, Sapanet M, Grévin G, Bailet P, Ollier A (1996) Identification by frontal sinus pattern in forensic anthropology. Forensic Sci Int 83:147–153. 10.1016/S0379-0738(96)02033-6 9022276 10.1016/s0379-0738(96)02033-6

[14] Lemos YV, Furtado AN, Lima AZ, Dionísio AS, Araújo RM, Cunha E (2024) Human identification by medical findings in a forensic anthropology context. Forensic Sci Res 9. 10.1093/fsr/owae041 10.1093/fsr/owae041 PMC11530380 39493280

[15] De Angelis D, Gibelli D, Palazzo E, Sconfienza L, Obertova Z, Cattaneo C (2016) Skeletal idiopathic osteosclerosis helps to perform personal identification of unknown decedents: A novel contribution from anatomical variants through CT scan. Sci Justice 56:260–263. 10.1016/j.scijus.2016.03.003 27320398 10.1016/j.scijus.2016.03.003

[16] Decker SJ, Ford JM (2019) Forensic personal identification utilizing part-to-part comparison of CT-derived 3D lumbar models. Forensic Sci Int 294:21–26. 10.1016/j.forsciint.2018.10.018 30445251 10.1016/j.forsciint.2018.10.018

[17] Palamenghi A, Cappella A, Cellina M, De Angelis D, Sforza C, Cattaneo C, Gibelli D (2023) Assessment of Anatomical Uniqueness of Maxillary Sinuses through 3D–3D Superimposition: An Additional Help to Personal Identification. Biology (Basel) 12:1018. 10.3390/biology12071018 37508447 10.3390/biology12071018 PMC10376834

[18] Gibelli D, Cellina M, Cappella A, Gibelli S, Panzeri MM, Oliva AG, Termine G, De Angelis D, Cattaneo C, Sforza C (2019) An innovative 3D-3D superimposition for assessing anatomical uniqueness of frontal sinuses through segmentation on CT scans. Int J Legal Med 133:1159–1165. 10.1007/s00414-018-1895-4 30039273 10.1007/s00414-018-1895-4

[19] Cappella A, Gibelli D, Cellina M, Mazzarelli D, Oliva AG, De Angelis D, Sforza C, Cattaneo C (2019) Three-dimensional analysis of sphenoid sinus uniqueness for assessing personal identification: a novel method based on 3D-3D superimposition. Int J Legal Med 133:1895–1901. 10.1007/s00414-019-02139-5 31396701 10.1007/s00414-019-02139-5

[20] Beaini TL, Duailibi-Neto EF, Chilvarquer I, Melani RFH (2015) Human identification through frontal sinus 3D superimposition: Pilot study with Cone Beam Computer Tomography. J Forensic Leg Med 36:63–69. 10.1016/j.jflm.2015.09.003 26408391 10.1016/j.jflm.2015.09.003

[21] Wang X, Wei S, Zhao Z, Luo X, Song F, Li Y (2024) 3D-3D superimposition techniques in personal identification: A ten-year systematic literature review. Forensic Sci Int 365:112271. 10.1016/j.forsciint.2024.112271 39476742 10.1016/j.forsciint.2024.112271

[22] Milheiro A, De Tobel J, Capitaneanu C, Shaheen E, Fieuws S, Thevissen P (2024) Quantifying the potential of morphological parameters for human dental identification: part 1—proof of concept. Int J Legal Med 138:25–34. 10.1007/s00414-022-02853-7 35704093 10.1007/s00414-022-02853-7

[23] Black S, MacDonald-McMillan B, Mallett X (2014) The incidence of scarring on the dorsum of the hand. Int J Legal Med 128:545–553. 10.1007/s00414-013-0834-7 23404533 10.1007/s00414-013-0834-7 PMC4008804

[24] Black S, MacDonald-McMillan B, Mallett X, Rynn C, Jackson G (2014) The incidence and position of melanocytic nevi for the purposes of forensic image comparison. Int J Legal Med 128:535–543. 10.1007/s00414-013-0821-z 23420260 10.1007/s00414-013-0821-z PMC4008801

[25] Cappella A, Solazzo R, Mazzarelli D, Gibelli D, Dolci C, Sforza C, Cattaneo C (2024) The potential of facial nevi in personal identification. Sci Rep 14:6206. 10.1038/s41598-024-56847-z 38485806 10.1038/s41598-024-56847-z PMC10940291

[26] Cappella A, De Angelis D, Mazzarelli D, Vitale A, Caccia G, Fracasso T, Cattaneo C (2022) Rediscovering the value of images in supporting personal identification of missing migrants. Leg Med 54:101985. 10.1016/j.legalmed.2021.101985 10.1016/j.legalmed.2021.101985 34753067

[27] Miranda GE, de Freitas SG, Maia LV, de Melani A RFH (2016) An unusual method of forensic human identification: use of selfie photographs. Forensic Sci Int 263:e14–e17. 10.1016/j.forsciint.2016.04.028 27138238 10.1016/j.forsciint.2016.04.028

[28] Palamenghi A, Gibelli D, Mazzarelli D, De Angelis D, Sforza C, Cattaneo C (2023) Rumor has it: A narrative review on the use of skeletal non-metric traits and variants for personal identification. Leg Med 65:102316. 10.1016/j.legalmed.2023.102316 10.1016/j.legalmed.2023.102316 37597346

[29] Verna E, Piercecchi-Marti M-D, Chaumoitre K, Adalian P (2015) Relevance of discrete traits in forensic anthropology: From the first cervical vertebra to the pelvic girdle. Forensic Sci Int 253. 10.1016/j.forsciint.2015.05.005. :134.e1-134.e7 10.1016/j.forsciint.2015.05.005 26048863

[30] Verna E, Piercecchi-Marti MD, Chaumoitre K, Bartoli C, Leonetti G, Adalian P (2013) Discrete Traits of the Sternum and Ribs: A Useful Contribution to Identification in Forensic Anthropology and Medicine. J Forensic Sci 58:571–577. 10.1111/1556-4029.12111 23550726 10.1111/1556-4029.12111

[31] Stephan CN, Winburn AP, Christensen AF, Tyrrell AJ (2011) Skeletal Identification by Radiographic Comparison: Blind Tests of a Morphoscopic Method Using Antemortem Chest Radiographs* ^,†,‡^. J Forensic Sci 56:320–332. 10.1111/j.1556-4029.2010.01673.x 21306373 10.1111/j.1556-4029.2010.01673.x

[32] Mann RW, Hunt DR (2019) Non-metric traits and anatomical variants that can mimic trauma in the human skeleton. Forensic Sci Int 301:202–224. 10.1016/j.forsciint.2019.05.039 31176138 10.1016/j.forsciint.2019.05.039

[33] Macaluso PJ, Lucena J (2014) Morphological variations of the anterior thoracic skeleton and their forensic significance: Radiographic findings in a Spanish autopsy sample. Forensic Sci Int 241. 10.1016/j.forsciint.2014.05.009. :220.e1-220.e7 10.1016/j.forsciint.2014.05.009 24933632

[34] Cunha E (2006) Pathology as a Factor of Personal Identity in Forensic Anthropology. In: Schmitt A, Cunha E, Pinheiro J (eds) Forensic Anthropology and Medicine. Humana, Totowa, NJ, pp 333–358

[35] Watamaniuk L, Rogers T (2010) Positive Personal Identification of Human Remains Based on Thoracic VertebralMargin Morphology. J Forensic Sci 55:1162–1170. 10.1111/j.1556-4029.2010.01447.x 20533977 10.1111/j.1556-4029.2010.01447.x

[36] Christensen AM, Hatch GM (2016) Quantification of radiologic identification (RADid) and the development of a population frequency data repository. J Forensic Radiol Imaging 7:14–16. 10.1016/j.jofri.2016.11.001

[37] Christensen AM, Hatch GM, Brogdon BG (2014) A current perspective on forensic radiology. J Forensic Radiol Imaging 2:111–113

[38] Scott S, Rogers TL (2026) Statistical support for identification using epigenetic traits of the human skeleton. Forensic Sci Int 378:112673. 10.1016/j.forsciint.2025.112673 41045694 10.1016/j.forsciint.2025.112673

[39] Palamenghi A, Borlando A, De Angelis D, Sforza C, Cattaneo C, Gibelli D (2021) Exploring the potential of cranial non-metric traits as a tool for personal identification: the never-ending dilemma. Int J Legal Med 135:2509–2518. 10.1007/s00414-021-02654-4 34275004 10.1007/s00414-021-02654-4 PMC8523454

[40] Palamenghi A, Aragon-Molina A, Caccia G, Mazzarelli D, Alemanno S, Donida Labati R, Scotti F, Piuri V, Campobasso CP, Cattaneo C, De Angelis D, Gibelli D (2025) From traditional to innovative: implications of cranial non-metric traits in personal identification. Int J Legal Med. 10.1007/s00414-025-03462-w 10.1007/s00414-025-03462-w PMC12170784 40029409

[41] Yushkevich PA, Piven J, Hazlett HC, Smith RG, Ho S, Gee JC, Gerig G (2006) User-guided 3D active contour segmentation of anatomical structures: Significantly improved efficiency and reliability. NeuroImage 31:1116–1128. 10.1016/j.neuroimage.2006.01.015 16545965 10.1016/j.neuroimage.2006.01.015

[42] de Boer HH, Obertová Z, Cunha E, Adalian P, Baccino E, Fracasso T, Kranioti E, Lefévre P, Lynnerup N, Petaros A, Ross A, Steyn M, Cattaneo C (2020) Strengthening the role of forensic anthropology in personal identification: Position statement by the Board of the Forensic Anthropology Society of Europe (FASE). Forensic Sci Int 315:110456. 10.1016/j.forsciint.2020.110456 32866741 10.1016/j.forsciint.2020.110456

[43] Aragon-Molina A, Alemanno S, Caccia G, Campobasso C, Cattaneo C, de Angelis D, Labati RD, Gibelli D, Palamenghi A, Piuri V, Scotti F (2025) Towards Automatic Computation of the SkullCode: A Novel Biometric Approach for Cranial Identification. In: 2025 IEEE International Conference on Computational Intelligence and Virtual Environments for Measurement Systems and Applications (CIVEMSA), Piraeus, Greece, pp. 1–6. 10.1109/CIVEMSA65862.2025.11084837

[44] Gibelli D, Cellina M, Gibelli S, Oliva AG, Termine G, Sforza C (2020) Are coding systems of frontal sinuses anatomically reliable? A study of correlation among morphological and metrical features. Int J Legal Med 134:1897–1903. 10.1007/s00414-020-02293-1 32279240 10.1007/s00414-020-02293-1

[45] Sagar S, Jahan S, Kashyap SK (2023) Prevalence of Anatomical Variations of Sphenoid Sinus and Its Adjacent Structures Pneumatization and Its Significance: A CT Scan Study. Indian J Otolaryngol Head Neck Surg 75:2979–2989. 10.1007/s12070-023-03879-y 37974780 10.1007/s12070-023-03879-y PMC10645943

[46] Carter LC, Pfaffenbach A, Donley M (1999) Hyperaeration of the sphenoid sinus: Cause for concern? Oral Surgery, Oral Medicine, Oral Pathology. Oral Radiol Endodontology 88:506–510. 10.1016/S1079-2104(99)70071-5 10.1016/s1079-2104(99)70071-5 10519764

[47] Batista Sandre L, Viandelli Mundim-Picoli MB, Fortes Picoli F, Rodrigues LG, Bueno JM, da Ferreira R (2017) Prevalence of agenesis of frontal sinus in human skulls with metopism. J Forensic Odontostomatol 35:20–27 29384733 PMC6100220

[48] Grine FE, Greening VA, Hernandez E, Billings BK, Mngomezulu V, Mongle CS (2024) Metopism in adult South Africans and its relationship to frontal sinus size. Anat Rec 307:2018–2035. 10.1002/ar.25350 10.1002/ar.25350 37955273

[49] Bilgin S, Kantarcı UH, Duymus M, Yildirim CH, Ercakmak B, Orman G, Gunenc Beser C, Kaya M, Gok M, Akbasak A (2013) Association between frontal sinus development and persistent metopic suture. Folia Morphol (Warsz) 72:306–310. 10.5603/FM.2013.0051 24402751 10.5603/fm.2013.0051

